# Abdominal actinomycosis misconceived as intestinal lymphoma: Report of a case

**DOI:** 10.1016/j.ijscr.2019.05.063

**Published:** 2019-06-08

**Authors:** Young-Hoon Roh, Ki-Jae Park, Kyung-Do Byun, Mee-Sook Roh, Hong-Jo Choi

**Affiliations:** a Department of Surgery, Dong-A University College of Medicine, Busan, Republic of Korea; b Department of Pathology, Dong-A University College of Medicine, Busan, Republic of Korea

**Keywords:** Actinomycosis, Intussusception, Lymphoma

## Abstract

**Introduction:**

Abdominal actinomycosis is a rare clinical entity and it is difficult to make the correct preoperative diagnosis because of its nonspecific clinical symptoms and varied radiological findings. The diagnosis is usually made after the patient undergoes an operation and tissue is available for pathological evaluation. When the diagnosis is made, the patient should be treated with the appropriate long-term antibiotics.

**Presentation of case:**

A 69-year-old male patient was transferred to our hospital complaining of a palpable mass, painful abdominal discomfort, weight loss, and night sweating. After colonoscopy and radiologic studies, our presumptive diagnosis was intestinal lymphoma combined with ileocecal intussusception. He was found to have abdominal actinomycosis after surgery and successfully treated with ampicillin for six months.

**Discussion:**

Pre-operative radiological imaging in abdominal actinomycosis is unlikely to allow a definitive diagnosis, but CT scanning is the single most useful imaging modality. Although we performed preoperative radiological studies, including CT, none led to a diagnosis of abdominal actinomycosis, we mistakenly considered the case as intestinal lymphoma combined with ileocecal intussusception.

**Conclusion:**

Physicians should include abdominal actinomycosis in the differential diagnosis when an abdominal mass presents an irregular, infiltrative growth pattern, even though ileocecal intussusception is an extremely rare presentation in abdominal actinomycosis.

## Highlights

• Diagnosis of abdominal actinomycosis is difficult because it is a rare entity with many different clinical features.
• Abdominal actinomycosis is one of the differential diagnoses of an abdominal mass and generally misconceived as a neoplasm.
• Ileocecal intussusception caused by abdominal actinomycosis is an extremely rare and unique presentation.
• Abdominal actinomycosis can be cured by prolonged antibiotic use with surgical resection.

## 1 Introduction

Abdominal actinomycosis is a chronic suppurative infectious disease caused by gram-positive *Actinomyces israelii* organisms. These organisms are not regarded as virulent pathogens but as opportunistic ones in humans, as they are normally present in healthy individuals. They become pathogens only in the presence of damaged or necrotic tissue; *Actinomyces* does not penetrate the normal mucosa and requires injury to cause disease [1,2].

Abdominal actinomycosis is rare and its variation makes diagnosis difficult. Computed Tomography (CT) shows a mass with an extensive infiltration pattern that closely resembles a complicated gastrointestinal malignancy. Also, the pattern of spread through contiguous tissues is not unlike inflammatory diseases [3]. Therefore, in patients with abdominal infiltrating masses, abdominal actinomycosis remains an important differential diagnosis from neoplasms, inflammatory bowel disease, diverticulitis, intestinal tuberculosis, and tubo-ovarian abscess [1,4]. It is well known that radiological signs of abdominal actinomycosis are diverse. However, ileocecal intussusception caused by abdominal actinomycosis is very rare. Here, we report a case of pathologically confirmed abdominal actinomycosis that was preoperatively considered as intestinal lymphoma with ileocecal intussusception, which presented unusual findings of radiological studies and colonoscopy.

This work has been reported in line with the SCARE criteria [5].

## 2 Presentation of case

A 69-year-old male patient was transferred to our hospital because of the palpable mass in the right lower quadrant of the abdomen that had been present for one month. He had been well until three months previously when he began to suffer from a poor oral intake, painful abdominal discomfort, general weakness, weight loss, and night sweating. Around 30 and 35 years before the current admission, the patient had been admitted for renal disease of unknown etiology and appendectomy for acute appendicitis, respectively. The body temperature was 36.8 °C. On physical examination, the patient appeared relatively well. A transverse operative wound for the previous appendectomy was identified in the right lower quadrant of the abdomen, and an adult-fist-sized mass was palpated without peritoneal irritation. Laboratory examination revealed mild leukocytosis of 14,000/mm^3^, normocytic normochromic anemia of 9.1 g/dl, elevated CRP to 15.98 mg/dl, elevated ESR to 120 mm/h, elevated serum creatinine and BUN to 2.9 mg/dl and 27 mg/dl each. Serum levels of AFP, CEA, and CA-19-9 were normal.

**Fig. 1:**
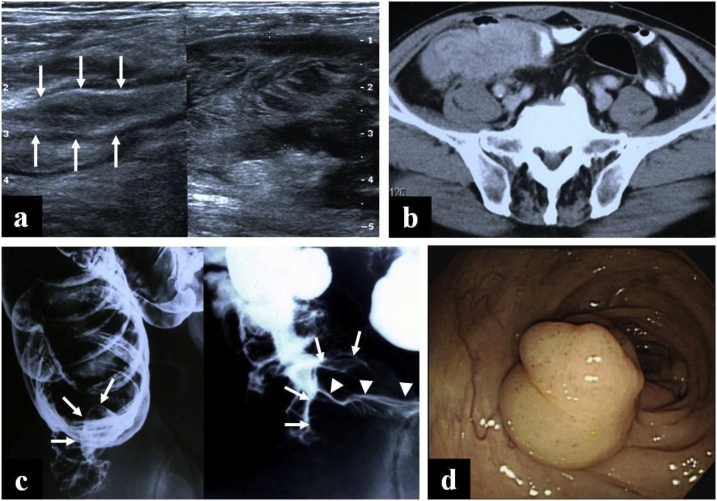
a. Ultrasonograms show intact mucosa and thickened submucosal layer with short segment intussusception (arrow) of the terminal ileum. b. Contrast enhanced CT scans represent homogeneous mural attenuation with mild pericolic infiltration. Note the short intussusception of terminal ileum. c. Colon contrast study: Short intussuscepted segment (arrow) of the terminal ileum was visualized in the cecum. Terminal ileum shows relatively intact mucosa (arrow head) and thickened fold. d. Colonoscopy shows a prominent protrusion of ileocecal valve.

Abdominal ultrasonography showed results that could not exclude distal ileal inflammatory disease or lymphoma (Fig. 1a). A contrast-enhanced CT revealed a terminal ileal mass with subsequent intussusception, which radiologically implied a lymphoma rather than inflammatory disease (Fig. 1b). A contrast study demonstrated an intussuscepted segment of the terminal ileum, and the mucosa was relatively intact (Fig. 1c). Colonoscopy demonstrated a prominent protrusion of the ileocecal valve (Fig. 1d). Examination of a specimen obtained by colonoscopic biopsy showed chronic inflammation with erosion.

**Fig. 2:**
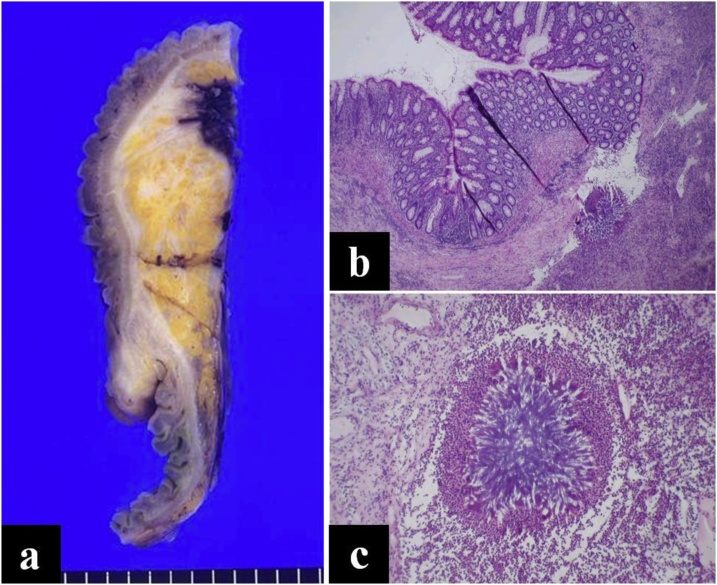
a. Gross finding of resected specimen. b. The section of the cecal wall shows a single, eosinophilic granule within abscess cavity (hematoxylin & eosin stain; ×40). c. The section of abscess cavity shows an amphophilic sulfur granule ensheathed by eosinophilic, serrate material (hematoxylin & eosin stain; ×100).

With a presumptive diagnosis of intestinal lymphoma, an exploratory laparotomy was performed. A large mass was located in the right lower abdomen, and it adhered densely to the retroperitoneum and the bladder. The mass including the right colon was mobilized from the retroperitoneum, avoiding injury of the right ureter. Multiple intraoperative pathological examinations of the mass and adhered retrocecal lesions by frozen-sections revealed chronic inflammation without any evidence of malignancy in the morphology. We performed the right hemicolectomy with drainage. The permanent pathological examination confirmed ileocecal actinomycosis in which the characteristic finding of sulfur granules in the resected specimen was demonstrated (Fig. 2). After surgery, the patient continued antibiotic treatment with ampicillin for six months and showed no signs of recurrence after two years.

## 3 Discussion

Actinomyces israelii is the most common organism in human actinomycosis. Actinomyces was initially thought to be a fungus but is now known to be a filamentous, non-spore-forming, gram-positive bacillus which is microaerophilic or obligate anaerobe. The general growth pattern is the direct extension of an existing lesion regardless of natural anatomic barriers, but hematogenous or lymphatic spread, although rare, can occur if organisms penetrate the damaged mucosa [6]. The pathogens produce a characteristic granulomatous inflammatory response which may cause multiple abscess formations, draining sinus, abundant granulation, extensive fibrosis, and a mass lesion [[7], [8], [9]].

Possible predisposing factors of disease progression include trauma, previous surgery, appendicitis, bowel perforation, foreign bodies including intrauterine devices (IUD), or neoplasia [10]. Among them, appendicitis, especially with perforation, is the most common predisposing condition. The ileocecal region, including the appendix, is known to be the most commonly involved area of the gastrointestinal tract [11]. Deshmukh et al. reported that two-thirds of 205 cases of abdominal actinomycosis involved the appendix and cecum [12]. Although the overall incidence of actinomycosis has decreased, the abdominopelvic form has been increasing because of the increased use of IUDs [8]. These etiological grounds may reflect the recent predominance of female abdominopelvic actinomycosis although no discernible gender difference has been reported [3,8,9].

Preoperative diagnosis of abdominal actinomycosis is rarely considered and is accurately established in less than 10% of cases. It is generally misconceived as a neoplasm, inflammatory bowel disease, diverticulitis, or tuberculosis both clinically and radiologically because of its characteristic infiltrative growth pattern [1,10]. Hence, actinomycosis has been called “the most misdiagnosed disease,” and there is “no disease which is so often missed even by experienced clinicians” [13]. There are myriad clinical manifestations of actinomycosis, and abdominal form is perhaps one of the greatest diagnostic challenges. Pre-operative radiological imaging is unlikely to allow a definitive diagnosis, but CT scanning is the single most useful imaging modality [3]. Although we performed preoperative radiological studies, including CT, none led to a diagnosis of abdominal actinomycosis. Interestingly, our case showed clinical manifestations similar to those of intestinal lymphoma and also presented prominent protrusion of the ileocecal valve. Thus, we mistakenly considered the case as intestinal lymphoma combined with ileocecal intussusception.

Definitive diagnosis of actinomycosis requires either isolation of the *A. israelii* in culture or demonstration of the specific “sulfur granules” microscopically. Unfortunately, the actinomycotic granules are found only in 50% of cases and routine bacteriological cultures of anaerobic organisms are negative for *A. israelii* [14]. Although it has been suggested that CT with fine needle aspiration may not only be diagnostic but may also be therapeutic, biopsies taken by aspiration are usually ineffective [4,10,14,15]. Therefore, although not specific, actinomycosis should be included in the differential diagnosis when CT scans show bowel wall thickening and a regional pelvic or peritoneal mass with an extensive infiltration pattern, especially in long-term IUD users or in patients with abdominal pain, fever, and leukocytosis, which are regarded as the most common constitutional symptoms of actinomycosis [3,4].

Increasing evidence suggests that medical therapy alone is usually sufficient to cure actinomycosis, including extensive invasive disease, if an accurate diagnosis is made before surgery [15]. Furthermore, if the correct diagnosis is made intraoperatively by frozen biopsy, the disease will be cured by a minimal extent of resection and antibiotics [8]. However, surgical excision of infected or necrotic tissue is generally recommended to reduce the bacterial burden and enhance the action of the antibiotics. Patients with actinomycosis require medication of penicillin G or amoxicillin for 6–12 months, but the duration of antimicrobial therapy could probably be shortened to three months in patients where optimal surgical resection of infected tissues has been performed [16]. We carried out the right hemicolectomy without complete resection of the retroperitoneal mass because multiple frozen biopsies showed chronic inflammation without evidence of malignancy. The permanent pathological findings showed typical “sulfur granules” compatible with actinomycosis and the patient was cured of the disease by a six-month administration of oral ampicillin, as evidenced by CT at the follow-up two years after surgery.

## 4 Conclusion

As described, the authors experienced an interesting case of abdominal actinomycosis combined with ileocecal intussusception. Ileocecal intussusception is an extremely rare presentation in abdominal actinomycosis. Nonetheless, physicians should include abdominal actinomycosis in the differential diagnosis when an abdominal mass presents an irregular, infiltrative growth pattern.

## Conflicts of interest

The authors declare that they have no conflict of interest.

## Sources of funding

None.

## Ethical approval

No research ethics approval was necessary for this case report.

## Consent

Written informed consent was obtained from the patient for publication of this case report and accompanying figures.

## Author contribution

Young-Hoon Roh, Kyung-Do Byun, Mee-Sook Roh, Hong-Jo Choi: Data collection, writing original draft.

Ki-Jae Park: study concept, methodology, writing reviewing and editing, advised and designed the report.

All authors read and approved the final manuscript.

## Registration of research studies

N/A.

## Guarantor

Ki-Jae Park.

## Provenance and peer review

Not commissioned, externally peer reviewed.
